# Improved Performance of Fundamental Mode Orthogonal Fluxgate Using a Micro-Patterned Meander-Shaped Ribbon Core

**DOI:** 10.3390/s19235058

**Published:** 2019-11-20

**Authors:** Shaotao Zhi, Zhu Feng, Chong Lei

**Affiliations:** Key Laboratory of Thin Film and Microfabrication Technology (Ministry of Education), Department of Micro/Nano Electronics, School of Electronic Information and Electrical Engineering, Shanghai Jiao Tong University, Dongchuan Road 800, Shanghai 200240, China; feng.z@sjtu.edu.cn

**Keywords:** orthogonal fluxgate, fundamental mode, meander-shaped core, micro-patterning, amorphous ribbon

## Abstract

In this paper, the performance of orthogonal fluxgate sensors with meander-shaped cores is studied in fundamental mode. The meander-shaped cores are made by micro-patterning technology based on a Co-based amorphous ribbon. The main advantage of this structure is that the linear operating range of the sensor can be adjusted simply by changing the number of strips, without affecting the excitation mechanism. Experiments show that a linear range of 560 μT is obtained by a meander-shaped core sensor with 12 strips. The changes in the number of strips can also increase sensitivity and reduce noise of the sensor. We can achieve a sensitivity of 600 V/T and a noise level of 0.64 nT/√Hz at 1 Hz for a meander-shaped core sensor with eight strips. Compared with the performance of the sensors built using a single strip core having the same equivalent cross-sectional area, the use of meander-shaped core can provide a higher sensitivity and linearity, and a lower noise level. We also compare the performance of an eight-strip meander-shaped core orthogonal fluxgate operated in the fundamental and second-harmonic modes. Similar sensitivity for the two modes can be obtained by adjusting the excitation current. In this case, we find that the noise of sensor operating in fundamental mode is about five times lower than that of the sensor operating in second-harmonic mode. This can be interpreted as the suppression of Barkhausen noise by unipolar bias in the fundamental mode.

## 1. Introduction

Fluxgate sensors are widely used vector magnetic field sensors used in measuring the dc or low-frequency ac magnetic fields [1]. High linearity, high temperature stability and room temperature operation characteristics of the fluxgate sensors enable them to be applied in many fields, such as electronic compasses [2,3], non-destructive testing [4], current sensors [5,6], bio-medical diagnostics [7,8,9] and space exploration [10].

Orthogonal fluxgate sensors have been attracting great interest due to their simplicity and miniaturization. The simplest orthogonal fluxgate is composed of a single magnetic core and a single sensing coil [11,12,13,14]. The excitation current is applied directly to the core and no excitation coil is needed. Furthermore, the noise of the orthogonal fluxgate can be strongly suppressed when the sensor operates in the fundamental mode [15,16,17,18,19,20]. In this mode, the core is kept continuously saturated due to the unipolar excitation current (ac excitation with large enough dc bias), and no reversal of the magnetization occurs.

In this paper, we suggest applying the fundamental mode operation to the orthogonal fluxgate with meander-shaped cores. The cores are prepared by micro-patterning technology with a Co-based amorphous ribbon. This material is chosen due to its high relative permeability, low coercivity and low magnetostriction. The designed meander-shaped core can improve the fluxgate performance by increasing the equivalent cross section of the sensing element without additional excitation current. On the other hand, it can reduce the demagnetization effect and eddy current effect as compared with increasing the width of the core directly.

The main goals of this work are as follows: (1) to investigate the effect of the number of strips for a meander-shaped core on the sensitivity, linear range and noise of sensor, (2) to compare the performance of the sensors constructed with the single strip and meander-shaped cores having the same length and equivalent cross-sectional area, and (3) to compare the performance of meander-shaped core orthogonal fluxgate sensor operated in the fundamental and in the second-harmonic modes.

## 2. Experimental Details

### 2.1. Micro-Patterned Core

**Figure 1:**
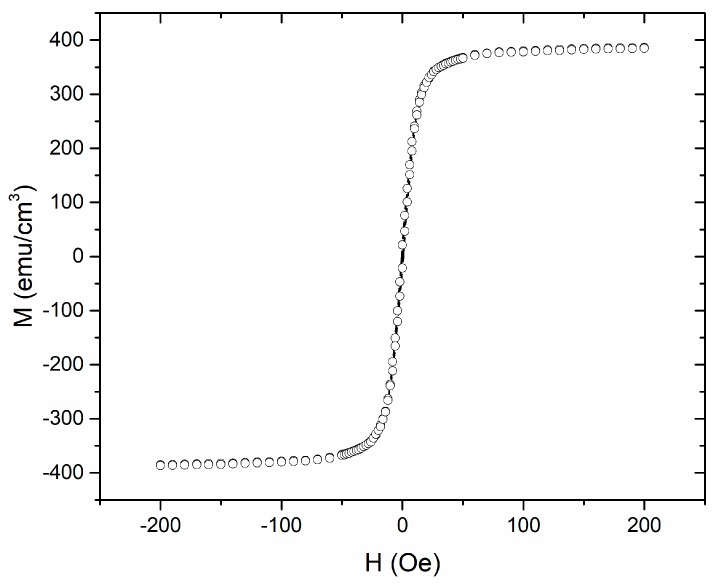
Hysteresis loop of the 2714A Co-based amorphous ribbon.

The orthogonal fluxgate cores were made of a Co-based commercial amorphous ribbon (Metglas 2714A, Metglas Inc., Hong Kong, China) with a thickness of 20 µm. The hysteresis loop of the ribbon sample was measured using a vibrating sample magnetometer (VSM), as shown in Figure 1. It shows nearly zero coercivity and remanence.

**Figure 2:**
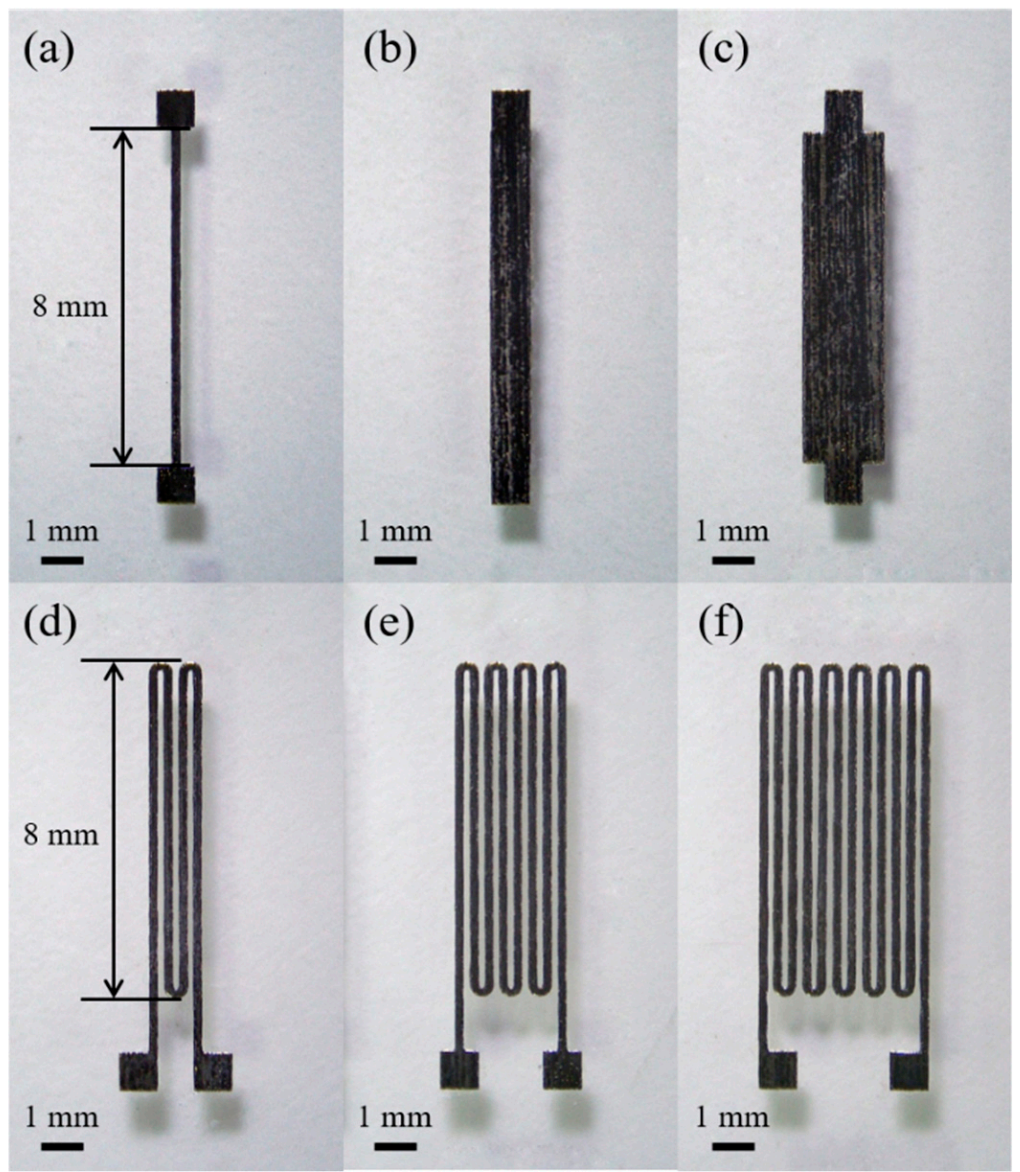
Photographs of the single strip cores with a width of (**a**) 200 µm, (**b**) 800 µm, (**c**) 1600 µm, and the meander-shaped cores with (**d**) 4, (**e**) 8, (**f**) 12 strips having the same strip width of 200 µm.

The micro-patterned ribbon cores with a single strip and meander-shaped structures are shown in Figure 2. The widths of single strip cores were 200, 800 and 1600 µm, respectively, and the meander-shaped cores were designed with strip width of 200 μm, interval of 150 μm, and the number of strips were 4, 8 and 12, respectively. In order to meet the requirements of sensor miniaturization, the length of all the strips was 8 mm. It should be noted that the meander-shaped cores have a rounded edge between the neighboring strips. This is because when the current passes through the core, the rounded edge has a more uniform magnetization at the corners compared to the square edge [21].

The fabrication process of the micro-patterned core samples was as follows: (1) the ribbon was bound on a clean glass substrate using an epoxy adhesive; (2) a thickness of 10 μm photoresist was spin-coated onto the surface of the ribbon and then patterned by UV lithography; (3) the ribbon was etched in an acidic mixed solution (HNO_3_:HCl:H_2_O_2_:H_2_O = 1:2:4:8) for about 5 min; (4) the photoresist was removed with an acetone solution, and the glass substrate was sliced into chips. A width error of ±2 μm was measured by a profiler and it was considered to be acceptable compared to the large size of the structures.

### 2.2. Measurement System

**Figure 3:**
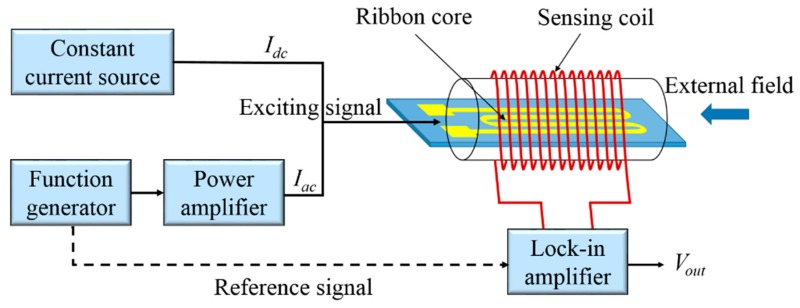
Schematic of micro-patterned ribbon core orthogonal fluxgate sensor and measurement system.

The experiment setup of the orthogonal fluxgate sensor employing micro-patterned ribbon cores with single strip and meander shape is shown in Figure 3. Sense winding having 100 turns, made from a 60 μm enameled copper wire, was wound on an acryl pipe of the outer diameter of 5 mm. The sensing coil with a length of 7 mm was located in the middle of the core, which was shorter than the core. This was because the terminations of the core did not add significant signal. The sinusoidal excitation ac current was supplied by a function generator (Tektronix AFG 3022, Tektronix, Beaverton, OR, USA) and amplified by a power amplifier (based on an OPA561, Texas Instruments, Dallas, Texas, USA). In the fundamental mode, an additional dc bias was required, which was provided by a constant current source (Instek PST 3202, GW Instek, Taiwan). The harmonic components of the output voltage were obtained using a lock-in amplifier (SR844, Stanford Research Systems, Sunnyvale, CA, USA). The external field was provided by a calibrated copper solenoid coil with a coil constant of 1.024 μT/mA and the intensity of the external field was determined by the Keithley 2450 source meter (Keithley Instruments, Cleveland, OH, USA). Both the sensors and the solenoid coil were placed inside a magnetic shielding cylinder, which consisted of six layers of soft magnetic ribbons, and the axis direction of the shield was perpendicular to the earth magnetic field.

## 3. Results and Discussion

### 3.1. Effect of the Number of Strips on the Performance

**Figure 4:**
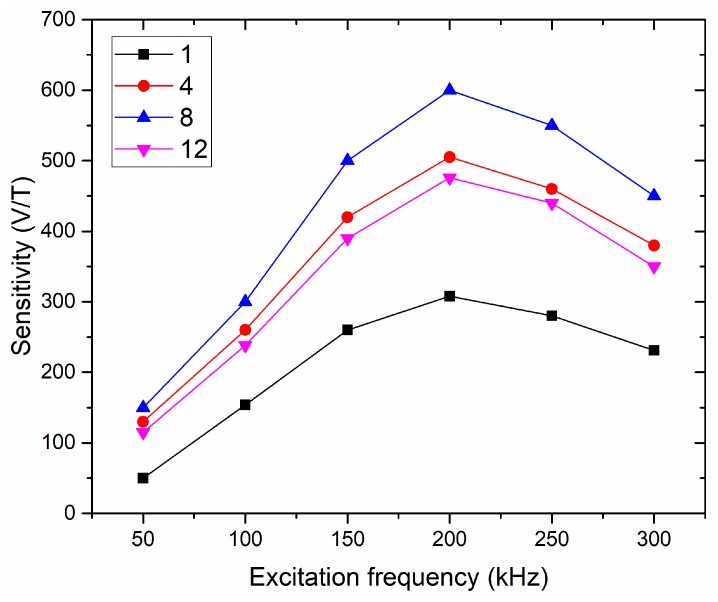
Dependence of the sensitivity of the sensor on the excitation frequency for the cores with one, four, eight and 12 strips.

We used the single strip and meander-shaped cores with four, eight and 12 strips having the same strip width of 200 µm as the core of the orthogonal fluxgate sensors. The sensors were operated in the fundamental mode, and the cores were excited by 150 mA ac current with 200 mA dc bias. Figure 4 shows the excitation frequency dependences of the sensor sensitivity for the cores with one, four, eight and 12 strips. The results show that the resonant frequency is independent of the number of strips in the cores, and it is always around 200 kHz for all the sensors. This is because the resonant frequency depends on the parasitic capacitance and inductance of the sensing coil [22], and the sensing coils in all the sensors are the same. Unless otherwise noted, the 200 kHz excitation frequency would be used for further experiments

**Figure 5:**
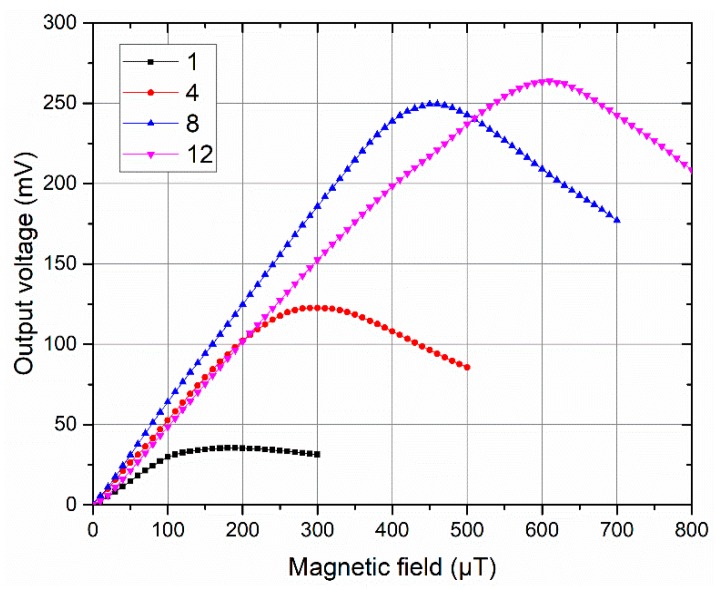
Responses of the sensors for the cores with one, four, eight and 12 strips.

**Figure 6:**
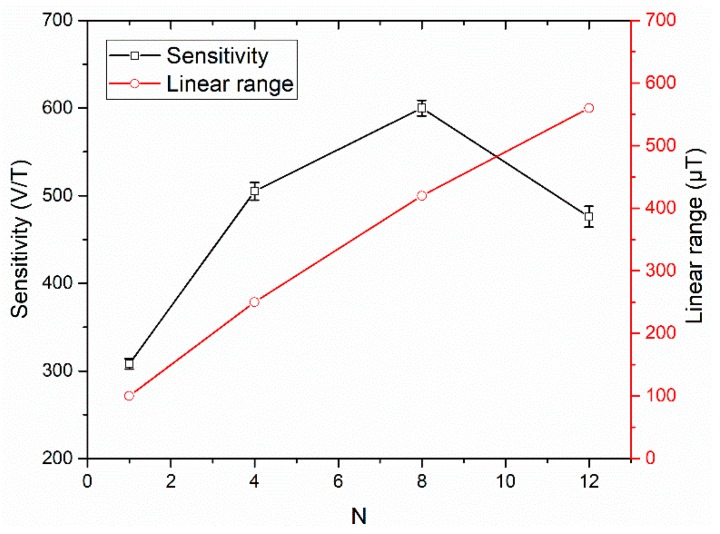
The sensitivity and linear range as a function of the number of strips.

Figure 5 shows the response curves of the sensors for the cores with one, four, eight and 12 strips. The linear operating range was calculated as the range in which the output response fitted to a linear function (R-squared value was greater than 99.6%), and the sensitivity of the sensor was the slope of the linear function. For the sensitivity data of each core five response curves were taken, and the sensitivity was the average value of the linear fit slopes and the error bars corresponded to the standard deviation. The sensitivity and linear range as a function of the number of strips are shown in Figure 6. The results show that with the increase of the strips, the sensitivity increases first and then decreases, and the linear operating range increases almost linearly. When the number of strips is 8, the sensitivity reaches its maximum value of 600 V/T, and a maximum linear range of 560 μT is obtained for the meander-shaped core sensor with 12 strips.

The reason for the variations in the sensitivity and linear range against the strip number increase can be understood by using the fluxgate output voltage equation. By taking demagnetization factor *D* into account, the induced voltage in the sensing coil can be calculated by the following equation [23]:

(1) $$V_{i}=-N_{2}\mu_{0}AH\frac{1-D}{{\left[1+D\left(\mu_{r}\left(t\right)-1\right)\right]}^{2}}\frac{d\mu_{r}\left(t\right)}{dt}$$

where *H* is the external field, *N*_2_ is the number of turns in the sensing coil, *A* is cross-sectional area of the magnetic core, *μ*_0_ and *μ_r_* are the vacuum and relative permeability, respectively.

Equation (1) clearly indicates that the output voltage can be generally increased by increasing the cross-sectional area of the core, but the limiting factor is the demagnetization factor. The demagnetization factor is related to the core geometry. In general, for a rectangular prism (in our case is the single strip core), the demagnetization factor is positively related to the aspect ratio of the core [24,25]. Therefore, when the core length is fixed, a wide core results in a large demagnetization factor. However, the calculation of demagnetization factor for a meander-shaped core is complex. We can approximate that the increase in the number of strips increases the overall width of core, which leads to an increase in the demagnetization factor. This can be proved from the sensitivity results of Figure 6. Due to the effect of the demagnetization, the sensitivity does not increase linearly with the increase of the number of strips, and the sensitivity increases slowly. When the number of strips is greater than 8, the sensitivity begins to decrease. Of course, compared with the increasing width of single strip core, the increasing number of strips in meander-shaped core has different effect on demagnetization factor. We will compare it in Section 3.2.

In addition, it should be noted that the linear range of the orthogonal fluxgate sensor increases with the increase of the demagnetization factor, which is due to the demagnetization effect that makes the apparent relative permeability of the core deviate from its intrinsic value [26], and it can be expected that the linear range will continue to increase as the number of strips increase. Of course, the sensitivity and core size should also be considered.

**Figure 7:**
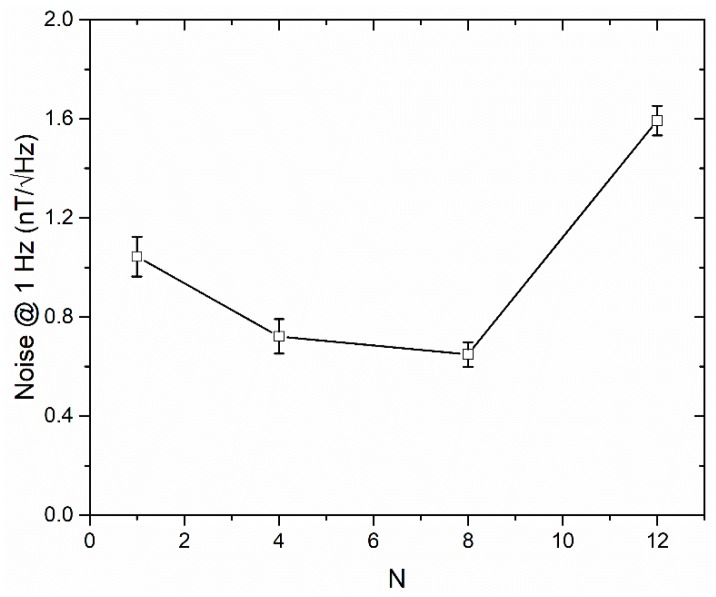
The noise at 1 Hz as a function of the number of strips.

The noise measurement was carried out in a magnetic shield, and a dynamic spectrum analyzer (Agilent 35670A, Agilent Technologies Inc., Palo Alto, CA, USA) was used to measure the noise spectra of the outputs from the lock-in amplifier. This voltage noise was then converted into the equivalent magnetic noise by dividing the value by the sensitivity at the very small region around the zero-field value. It is important to note that due to the good linearity of the sensor, the sensitivity at this region is similar to that over the whole linear range. Figure 7 shows the sensor noise values measured at 1 Hz for the cores with different number of strips. The results show that the eight-strip meander-shaped core has the lowest noise level due to the highest sensitivity, and the noise is 0.64 nT/√Hz measured at 1 Hz. Moreover, magnetic interactions between the strips of the core under the high-frequency magnetization by the current passing through it will also increase the noise of the sensor. Therefore, the noise of the 12-strip core was significantly increased.

### 3.2. Comparison of the Single Strip and Meander-Shaped Cores

**Figure 8:**
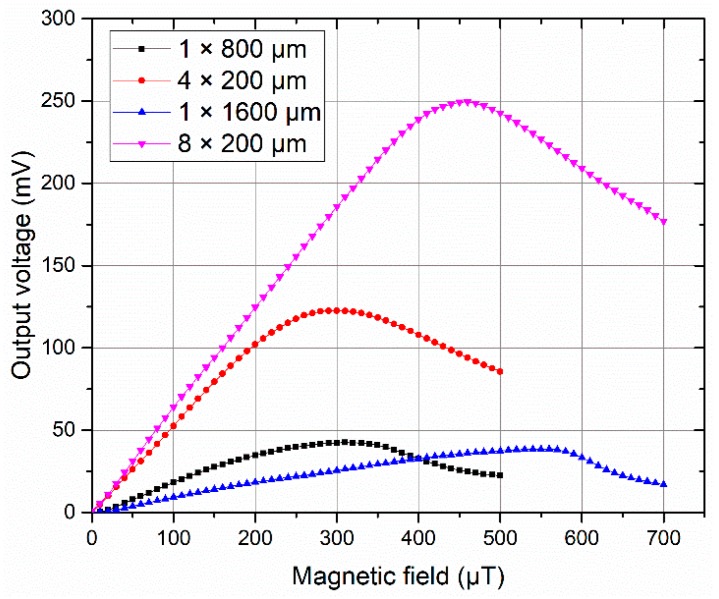
Comparisons of the output responses of the sensors for single strip and meander-shaped cores.

Two sets of cores were used to compare the performance differences of the sensors for the single strip and meander-shaped structure when the cross-sectional areas of the sensing elements were kept equal. The first test compared a single strip core of width 800 µm (1 × 800 µm) against a meander-shaped core with four strips (4 × 200 µm). The second test compared a single strip core of width 1600 µm (1 × 1600 µm) against a meander-shaped core with eight strips (8 × 200 µm). Figure 8 shows the comparisons of the output responses of the sensors for single strip and meander-shaped cores.

Obviously, the sensitivity and linearity of the input–output characteristics of the meander-shaped core sensors are better than that of the single strip core sensors. The four-strip meander-shaped core sensor shows the sensitivity of 505 V/T against 170 V/T in 800 µm width single strip core sensor, which is about three times higher, and the eight-strip meander-shaped core sensor shows the sensitivity of 600 V/T against 84 V/T in 1600 µm width single strip core sensor, which is about 7 times higher. The sensitivity comparison results indicate that the increasing demagnetization factor caused by the increase of strips for meander-shaped core is much smaller than that caused by the increase of the width for the single strip core under the same cross-sectional area.

**Figure 9:**
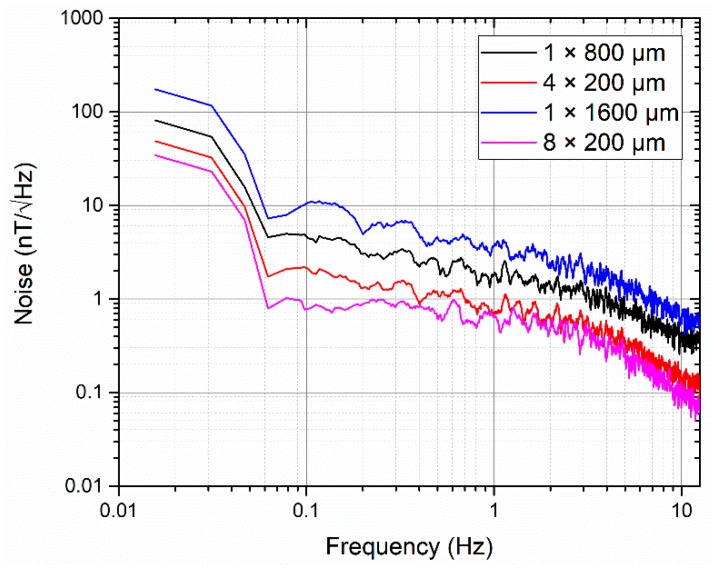
Comparisons of the noise spectrums of the sensors for single strip and meander-shaped cores.

As shown in Figure 9, the noise spectrums of the four sensors present the usual 1/f behavior, and the meander-shaped core sensors show a lower noise level compared with the single strip core sensors (0.72 nT/√Hz vs. 1.88 nT/√Hz at 1 Hz in the 4 × 200 µm core case vs. 1 × 800 µm core case, and 0.64 nT/√Hz vs. 3.67 nT/√Hz at 1 Hz in the 8 × 200 µm core case vs. 1 × 1600 µm core case).

### 3.3. Comparison of Fundamental and Second-Harmonic Modes

**Figure 10:**
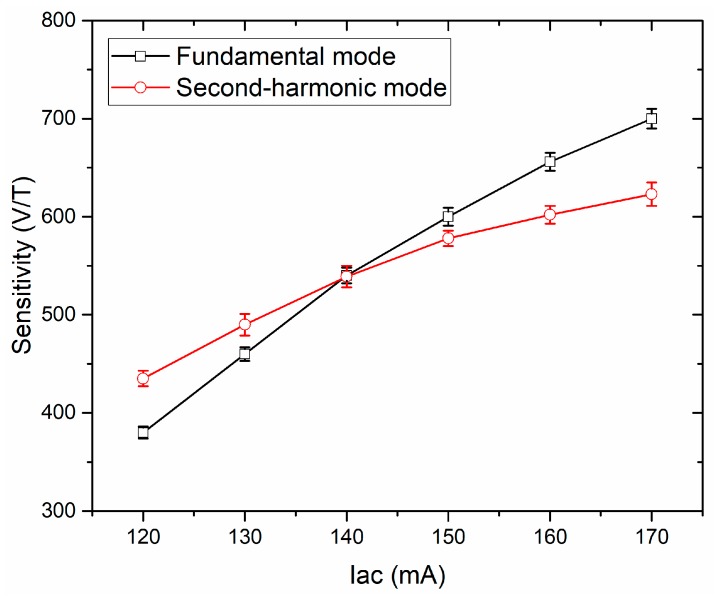
Dependence of sensitivity on ac current for an eight-strip meander-shaped core sensor working in fundamental mode (200 mA dc bias) and second-harmonic mode (without dc bias).

Figure 10 shows the dependence of sensitivity on ac current for the eight-strip meander-shaped core orthogonal fluxgate sensor working in the fundamental mode and second-harmonic mode. The excitation frequency of ac current was 200 kHz. In both modes, the sensitivity of the sensor increases with the increase of excitation ac current. When the ac current is greater than 140 mA, the sensitivity of the sensor in the fundamental mode is higher than that in the second-harmonic mode. This result is different from [15] and [17], which the sensitivity of the fundamental mode is always higher. This may be the consequence of the different materials and geometries used for the sensing elements. It is noted that in the fundamental mode, the sensitivity is also dependent on the dc bias, which is much larger than the amplitude of ac current. For a given ac current, the sensitivity drops as the dc bias current increases [27]. Moreover, the choice of dc bias will affect the noise of the sensor. In general, the large dc bias can suppress Barkhausen noise due to the continuous saturation of the core [18,20,28]. However, excessive dc bias makes the sensitivity decrease rapidly, which leads to the decrease of the total noise. In our case, in order to obtain a high sensitivity and a low noise, the 200 mA dc bias current is an optimized choice.

**Figure 11:**
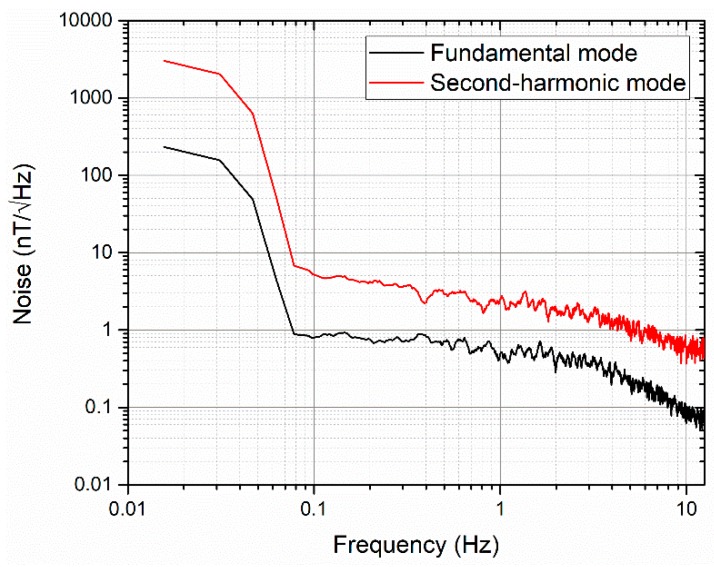
Noise spectrums of an eight-strip meander-shaped core sensor working in fundamental mode and second-harmonic mode.

From Figure 10, we can also see that when the ac current is 140 mA (for the second harmonic mode, equivalent to a current of 90 mA rms), the sensitivity of the two modes is similar, about 540 V/T. In such an excitation current, we compare the noise spectral density of the orthogonal fluxgate sensor working in fundamental mode and second-harmonic mode, as shown in Figure 11. We find that the noise spectral density at 1 Hz in the fundamental mode (0.50 nT/√Hz) is by a factor of 5 lower than that in the second-harmonic mode (2.49 nT/√Hz).

Although the noise of the sensor operating in fundamental mode is greatly suppressed, the noise level at 1 Hz is still higher than the noise level (2.5 pT/√Hz) of wire-core orthogonal fluxgate [29]. Two factors need to be considered. On the one hand, the center of the ribbon core is usually unsaturated. This is due to the fact that the circumferential field generated by the excitation current is not constant at every distance from the center to the edge of the core. According to Ampere’s law, the circular magnetic field rises linearity from zero at the center to the maximum at the edge of the core. Therefore, in the center of the core there will be a portion of the core which is not saturated. On the other hand, due to the wet etching process, there is a slight unevenness at the strip edge of the micro-patterned meander-shaped core, which is also a noise source that causes the Barkhausen effect. These need to be further improved in the later experiments.

## 4. Conclusions

The meander-shaped core orthogonal fluxgate sensor operating in fundamental mode shows excellent performance such as high sensitivity, good linearity and wide linear range. The experimental results show that the linear range of the meander-shaped core sensor can be arranged simply by changing the number of strips, and a sensitivity of 600 V/T and a noise level of 0.64 nT/√Hz at 1 Hz can be obtained for an eight-strip meander-shaped core sensor working in fundamental mode. When the equivalent cross-sectional area of the sensing element is equal, the meander-shaped core sensor exhibits a higher sensitivity and a lower noise compared with the single strip core sensor. For the same meander-shaped core, the noise in fundamental mode is reduced by a factor of 5 compared to that in the second-harmonic mode. In addition, the micro-patterned ribbon core meets the requirements for miniaturization and can be used to fabricate the Micro-Electro-Mechanical System (MEMS) sensors, and it is very suitable for the fabrication of biosensor array for measuring biological magnetic field. It needs further design and will be the subject of our future work.
